# Measuring serum matrix metalloproteinase-9 levels in peripheral blood after subarachnoid hemorrhage to predict cerebral vasospasm

**DOI:** 10.1186/s40064-016-2837-6

**Published:** 2016-07-22

**Authors:** Aykut Akpinar, Necati Ucler, Uzay Erdogan, Serhat S. Baydin, Abuzer Gungor, Bekir Tugcu

**Affiliations:** Department of Neurosurgery, Adiyaman University Education and Research Hospital, 02200 Adiyaman, Turkey; Bakirkoy Research and Training Hospital for Neurology Neurosurgery and Psychiatry, Istanbul, Turkey; Department of Neurosurgery, Mcknight Brain Institute, University of Florida, Gainesville, FL USA

**Keywords:** Matrix metalloproteinase-9, Ischemia, Subarachnoid hemorrhage, Aneurysm

## Abstract

**Purpose:**

We aimed to investigate serum levels of matrix metalloproteinase-9 in both subarachnoid hemorrhage and control groups for prediction of cerebral vasospasm in this study.

**Methods:**

Venous serum matrix metalloproteinase-9 levels were prospectively measured four times (days 1, 3, 7, and 14) for 34 consecutive patients with subarachnoidal hemorrhage (n = 27) and for elective aneurysm clipping (control, n = 7).

**Results:**

Vasospasm developed in 11/34 (32.4 %) patients between 3 and 10 days after subarachnoid hemorrhage (median 5.58 days), mean peak serum matrix metalloproteinase-9 compared with the non-vasospasm cohort. Matrix metalloproteinase-9 levels were higher in subarachnoid hemorrhage patients than in the controls.

**Conclusion:**

Increased serum matrix metalloproteinase-9 could be an accurate biomarker to predict the onset of cerebral vasospasm after subarachnoid hemorrhage.

## Background

Subarachnoid hemorrhage (SAH) is a stroke subtype cause by blood leakage from a ruptured intracerebral aneurysm. It is characterized by sudden onset and is associated with high morbidity and mortality, often attributable to cerebral vasospasm (VS) with secondary cerebral ischemia (Dorsch 1995; Horstmann et al. 2006).

Delayed cerebral VS as defined angiographically occurs in up to 70 % of patients who present with SAH and leads to delayed ischemic deficits for 36 % of patients (Biller et al. 1988). Matrix metalloproteinases (MMPs) can degrade extracellular matrix components in a variety of physiological and pathophysiological conditions such as stroke, intracerebral hemorrhage, and intracerebral aneurysms (Montaner et al. 2001; Todor et al. 1998).

In this study, we aimed to determine the reliability of MMP-9 measurements in patients with acute SAH and patients without SAH but with incidental aneurysms, and also we investigated whether MMP-9 levels were related to SAH severity or VS occurrence and aneurysms.

## Results

The clinical presentation [Hunt–Hess grade, Glasgow coma scale (GCS)] and radiological characteristics (Fisher grade, foci of hemorrhage) of 34 patients admitted with or without SAH are shown in Table 1.

**Table 1:** Patient’s admission and discharge scores and aneurysmal features

Patients’GCS at admission	15	n = 24 (70.6 %)
	14–10	n = 3 (8.7 %)
	9–3	n = 7 (20.7 %)
Patients’GCS at discharge	15	n = 27 (79.4 %)
	14–10	n = 3 (8.8 %)
	9–3	n = 4 (11.8 %)
Patients’ Fischer grade at admission	1 (no blood)	n = 9 (26.5 %)
	2 (less than 1 mm)	n = 6 (17.6 %)
	3 (more than 1 mm)	n = 12(35.3 %)
	4 (intracerebral or ventricular hemorrhage	n = 7 (20.6 %)
Patients’ Hunt Hess grade at admission	1	n = 20 (58.84 %)
	2	n = 6 (17.6 %)
	3	n = 4 (11.7 %)
	4/5	n = 4 (11.7 %)
Foci hemorrhage	Middle cerebral artery anevrsym	n = 11 (32.4 %)
	Anterior communican artery anevrsym	n = 8 (23.5 %)
	Posterior comminican anevrsym	n = 4 (11.8 %)
	Basilar type anevrsym	n = 1 (2.9 %)
	Carotid anevrsym	n = 1 (2.9 %)
	Arteriovenous malformation	n = 3 (8.8 %)
	Patients can not found the source of bleeding	n = 6 (17.6 %)
The numbers of aneurysyms	Single aneurysyms	19 (55.9 %)
	Two aneurysyms	4 (11.8 %)
	Three or more	2 (5.9 %)
	AVM	3 (8.8 %)
	No source of bleeding [but SAH (+)]	6 (17.6 %)

MMP-9 levels were significantly elevated in SAH group (27 patients) relative to the controls (7 patients). We measured MMP-9 levels in 27 SAH patients categorized by the presence or absence of VS as well as Hunt–Hess, Fisher, and GCS grades at admission. The overall pattern of higher MMP-9 levels was apparent in subjects with VS and a higher Hunt–Hess score. MMP-9 levels remained substantially above the mean control level until day 14, when they tended to decrease.

Overall, 11 (32.4 %) of total 34 patients developed VS. Eight patients with SAH required emergency surgery. Two patients were treated with medical therapy, and 1 patient who underwent elective aneurysm clipping developed VS.

Twenty-four (70.6 %) patients were discharged with normal neurological examination results. Ten (29.4 %) patients had neurological deficits [hemiparesis and ptosis (n = 6), and exitus (n = 4)], and 11 (32.4 %) patients developed VS. Clinical deterioration began between days 3 and 10 (median 5.58 days). A Chi square test revealed a significant interaction between the amount of blood in SAH (Fisher grade) and the development of VS (*p* = 0.035).

**Fig. 1:**
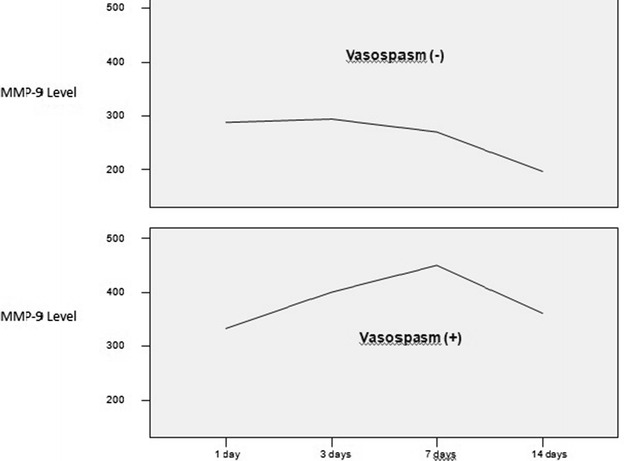
MMP-9 levels in the SAH group were higher than in the no-bleeding group, but this difference was not significant (*p* > 0.05)

MMP-9 levels were higher in the SAH group compared to the control group, but this was not significant (*p* > 0.05). The same was true for the higher MMP-9 levels in the VS group compared to the non-VS group (*p* > 0.05; Fig. 1).

## Discussion

MMP-9 plays an important role in ischemic and hemorrhagic stroke, and serum levels significantly increase after SAH. MMP-9 is known to be secreted as an inactive preform that is quickly degraded after activation. In the present study, we attempted to demonstrate that serum MMP-9 concentrations can effectively predict the onset of delayed cerebral VS several days before TCD velocity changes or neurological deterioration.

There is no single treatment for VS because of its multifactorial etiology. The aim of treatment is to prevent the development of VS and protect the brain against ischemia. Zhang et al. (2015) showed in rats that neurovascular protection of astaxanthin in SAH is partly associated with the inhibition of MMP-9 expression and activity. In this respect, MMP-9 inhibition may help prevention of VS in human SAH.

Lago et al. (2015) reported in the recent study that the infarcts were associated to SAH severity, SAH outcome and symptomatic vasospasm, and also metalloproteinase-9 was higher in SAH patients than in controls, but it could not discriminate the infarct patients. Severity of the initial insult, clinical status at admission, and Fisher grade were associated with increased MMP-9 concentration in our study. Patients who developed cerebral VS exhibited higher Hunt–Hess grade and Fisher grade, which is in agreement with an other study (Fisher et al. 1980).

Patients with higher Hunt–Hess scores on admission were more likely to be discharged with neurological deficits (*p* = 0.016). MMP-9 levels were higher in the SAH group compared to the control group. Egashira et al. (2015) reported that SAH causes blood brain barrier disruption and consequent injury in white matter. MMP-9 plays an important role in those pathologies and could be a therapeutic target for SAH-induced white matter injury.

Overall, 11 patients (32.4 %) developed VS. Clinical deterioration began between the third and tenth days (mean 5.58 days). Among 18 patients who had SAH aneurysm due to clipping, 8 patients experienced VS. Nine patients without operation [SAH (+)] were followed up medically, and two developed VS. One patient from the elective group experienced VS (surgical morbidity). Four (14.81 %) patients in the SAH group died. These findings are consistent with what is reported in the literature (Awad et al. 1987; Muizelaar and Becker 1986).

The identification of patients with delayed cerebral VS before clinical deterioration might allow aggressive and selective prophylactic intervention that could improve efficacy and minimize therapeutic complications and morbidity. Neurological recovery and improved outcomes associated with endovascular angioplasty or hypertensive, hemodilutional, or hypervolemic therapy after delayed cerebral VS might be dependent on early intervention (Medlock et al. 1992; Origitano et al. 1990).

The role of MMP-9 in VS is not clear (Minami et al. 1991). We rely on TCD and angiographic findings to measure increased velocities or identify stenotic lesions, respectively. However, TCD and angiographic results do not predict VS. The identification of a biomarker to predict VS would represent a significant advance in the field, allowing appropriate targeting of therapeutic prophylactic measures.

## Conclusion

Additional studies with larger numbers of patients who are treated several days earlier are needed to confirm the treatment of preclinical VS. This small prospective study indicates that MMP-9 might be a useful biomarker for identifying pre-ischemic delayed cerebral VS after SAH. Specifically, high serum MMP-9 concentrations may independently predict the onset of VS several days before ischemic deficits, potentially allowing accelerated angiographic evaluation and aggressive prophylactic intervention.

## Methods

We analyzed serum samples of SAH patients seen at our department between January 2012 and July 2013. The study was approved by the ethics committee of Bakirkoy Psychiatric and Neurological Research and Training Hospital, and all patients or their relatives gave informed consent. Patients’ characteristics were recorded, including age, sex, and the presence of diabetes mellitus and hypertension (Table 2).

**Table 2:** Demogrphic features of SAH group, control group and treatment of patients

Male (%)	8 (52.9)
Female (%)	6 (47.1)
Mean age	53.46 (26–77)
Hypertension (%)	15 (44.1)
Diabetes mellitus (%)	7 (20.6)
Vasospasm (%)	11 (34.1)
Subarachnoid hemorrhage	27 (79.4%)
Elective aneurysms clipping (without SAH)	7 (20.6 %)
Aneursymal clipping	25
Endovasculer coilling	1
Medical therapy	9
Patients with SAH without aneurysyms	6

Consecutive patients admitted with or without aneurysmal SAH (n = 27) or admitted for elective aneurysm clipping (n = 7) were enrolled. We planned to measure MMP-9 levels on days 1, 3, 7, and 14, which should span the period of increased VS risk.

All patients underwent TCD evaluations three times each week. The development of cerebral VS was confirmed angiographically by an independent neuroradiologist in patients who demonstrated a mean anterior cerebral artery or middle cerebral artery TCD velocity of >150 cm/s, or who exhibited neurological deficit onset after SAH.

Patients with aneurysm identified by angiography underwent surgery. Their risk factor profiles and Hunt–Hess score at admission were ascertained. The occurrence of VS was determined by TCD or the onset of new focal neurological deficits.

Collected blood samples were centrifuged (4000×*g*), and the resulting supernatants were immediately frozen at −20 °C until analysis. Commercially available enzyme-linked immunosorbent assay kits were used for quantitative determination of MMP-9 (Bender MedSystem, Vienna, Austria).

### Statistical methods

We examined the reliability of MMP-9 determinations from the replicate samples. We calculated MMP-9 levels in all patients between days 1 and 14 (1, 3, 7, and 14). We used the exact Mann–Whitney distribution to establish whether the MMP levels were significantly between SAH patients and those patients without incidental aneurysm.

Correlations between Fisher grade and serum MMP-9 levels were determined with multivariate logistic regression analyses, adjusting for patient age, sex, Hunt–Hess grade, Fisher grade, and GCS at admission.

## Abbreviations

SAH: subarachnoid hemorrhage
GCS: Glasgow coma scale
MMPs: matrix metalloproteinase
TCD: transcranial Doppler
VS: vasospasm
